# Inter-rater reliability and concurrent validity of ROBINS-I: protocol for a cross-sectional study

**DOI:** 10.1186/s13643-020-1271-6

**Published:** 2020-01-13

**Authors:** Maya M. Jeyaraman, Rasheda Rabbani, Nameer Al-Yousif, Reid C. Robson, Leslie Copstein, Jun Xia, Michelle Pollock, Samer Mansour, Mohammed T. Ansari, Andrea C. Tricco, Ahmed M. Abou-Setta

**Affiliations:** 1 The George and Fay Yee Center for Healthcare Innovation, University of Manitoba, 753 McDermot Avenue, Winnipeg, MB R3E 0T6 Canada; 2 Department of Community Health Sciences, University of Manitoba, Winnipeg, Canada; 3 Li Ka Shing Knowledge Institute, St. Michael’s Hospital, Unity Health Toronto, Toronto, Canada; 4 Nottingham Ningbo GRADE Centre, The University of Nottingham Ningbo, Ningbo, China; 5 Institute of Health Economics, Edmonton, Alberta Canada; 6 Centre Hospitalier de l’Université de Montreal, Quebec, Montreal Canada; 7 Faculty of Medicine, Department of Medicine, Université de Montréal, Quebec, Montreal Canada; 8 Centre de recherche du Centre Hospitalier de l’Université de Montréal, Quebec, Montreal Canada; 9 School of Epidemiology and Public Health, Faculty of Medicine, University of Ottawa, Ottawa, Canada; 10 Epidemiology Division, Dalla Lana School of Public Health and Institute of Health, Management, and Policy Evaluation, University of Toronto, Toronto, Canada; 11 Queen’s Collaboration for Health Care Quality Joanna Briggs Institute Centre of Excellence, Queen’s University, Kingston, Ontario Canada

**Keywords:** Inter-rater reliability, Inter-consensus reliability, Concurrent validity, ROBINS-I, Cross-sectional study, Non-randomized studies

## Abstract

**Background:**

The Cochrane Bias Methods Group recently developed the “Risk of Bias (ROB) in Non-randomized Studies of Interventions” (ROBINS-I) tool to assess ROB for non-randomized studies of interventions (NRSI). It is important to establish consistency in its application and interpretation across review teams. In addition, it is important to understand if specialized training and guidance will improve the reliability of the results of the assessments. Therefore, the objective of this cross-sectional study is to establish the inter-rater reliability (IRR), inter-consensus reliability (ICR), and concurrent validity of ROBINS-I. Furthermore, as this is a relatively new tool, it is important to understand the barriers to using this tool (e.g., time to conduct assessments and reach consensus—evaluator burden).

**Methods:**

Reviewers from four participating centers will appraise the ROB of a sample of NRSI publications using the ROBINS-I tool in two stages. For IRR and ICR, two pairs of reviewers will assess the ROB for each NRSI publication. In the first stage, reviewers will assess the ROB without any formal guidance. In the second stage, reviewers will be provided customized training and guidance. At each stage, each pair of reviewers will resolve conflicts and arrive at a consensus. To calculate the IRR and ICR, we will use Gwet’s AC_1_ statistic.

For concurrent validity, reviewers will appraise a sample of NRSI publications using both the New-castle Ottawa Scale (NOS) and ROBINS-I. We will analyze the concordance between the two tools for similar domains and for the overall judgments using Kendall’s tau coefficient.

To measure the evaluator burden, we will assess the time taken to apply the ROBINS-I (without and with guidance), and the NOS. To assess the impact of customized training and guidance on the evaluator burden, we will use the generalized linear models. We will use Microsoft Excel and SAS 9.4 to manage and analyze study data, respectively.

**Discussion:**

The quality of evidence from systematic reviews that include NRS depends partly on the study-level ROB assessments. The findings of this study will contribute to an improved understanding of the ROBINS-I tool and how best to use it.

## Background

Systematic reviews provide the best available evidence to inform healthcare decision-making [1]. The strength, or quality, of the evidence from systematic reviews depends partly on the internal validity of the included studies [2]. Evidence from randomized controlled trials (RCT) is superior to evidence originating from non-randomized studies (NRS) due to potential biases associated with the design and conduct of NRS [2]. Even so, it is important to include NRS in systematic reviews when evidence from RCTs is indirect, imprecise, inconsistent, inapplicable, or unavailable [3, 4]. As such, reviewers must be aware of the potential biases due to the design and conduct of NRS and the best practices to minimize the impact of these biases on the effect estimate [5].

Many quality assessment tools are available to assess the methodological quality of NRS [6–8]. Although unpublished to this day, the Newcastle-Ottawa scale (NOS) is the most widely used quality assessment tool for NRS [7]. NOS is eight items divided into three domains (selection, comparability, and either outcome or exposure assessment for cohort and case-control studies, respectively). Reviewers rate study quality using a star system with a maximum of one star assigned to all eight items (except for one item under comparability domain, which receives a maximum of two stars); total scores can range between 0 and 9 stars [7]. Although the NOS is widely used, it was reported to have poor inter-rater reliability [5].

Furthermore, in recent years, as our understanding of the potential effects of study design, study conduct, and study reporting has improved, there has been a major shift from using checklists for assessing study quality (or just reporting per se) to assessing ROB [9, 10]. While it may be counterintuitive, study quality, reporting, and risk of bias are not synonymous with each other; well-designed and conducted trials may be poorly reported and not all biases will relate to poor study quality and vice versa.

Over the years, researchers have developed hybrid ROB assessment tools by modifying existing available instruments. In 2014, the Cochrane Bias Methods Group developed “A Cochrane Risk Of Bias Assessment Tool: for Non-Randomized Studies of Interventions (ACROBAT-NRSI) [11]. This initial version of the tool was finalized and renamed the “Risk of Bias in Non-randomized Studies of Interventions” (ROBINS-I) in 2016 [12]. The ROBINS-I guides judgment about the ROB in an estimate of (a beneficial or harmful) effect of an intervention investigated in a NRS of interventions (NRSI) from the perspective of a hypothetical target RCT that the NRS best emulates (even if the RCT would be unethical or unfeasible) [2, 12]. The ROBINS-I tool is composed of seven domains to assess bias due to confounding, selection of participants, classification of interventions, departures from intended interventions, missing data, measurement of outcomes, and selection of reported results [12]. Each of the seven domains contains multiple signaling questions with five response options (*yes, probably yes, no, probably no, or no information*) to guide domain level ROB adjudications [12]. The ROB adjudications are categorized as follows: *low risk*, *moderate risk*, *serious risk*, *critical risk*, *or no information*. Although the use of ROBINS-I tool is currently very limited, it is expected to steadily increase with time.

Since ROBINS-I is a relatively new instrument, it is important to assess its psychometric properties. It is highly essential to establish ample evidence on its reliability and validity in order to assess and improve the consistency in its application and in how it is interpreted across various systematic reviews that include evidence from NRSI. Inter-rater reliability (IRR) refers to the reproducibility or consistency of decisions between two reviewers and is a necessary component of validity [13, 14]. Inter-consensus reliability (ICR) refers to the comparison of consensus assessments across pairs of reviewers in the participating centers. Concurrent validity refers to the extent to which the results of the instrument or tool can be trusted [14]. Furthermore, it is important to understand the barriers to using this tool (e.g., time to conduct assessments and reach consensus—evaluator burden).

## Methods/design

An international team of experienced researchers from four participating centers will collaboratively undertake this study. The major objectives are to

I. Measure the IRR and ICR between reviewers when assessing ROB of NRSI using ROBINS-I (without and with customized training and guidance);
II. Measure the concurrent validity of ROBINS-I;
III. Measure the evaluator burden (time taken to apply ROBINS-I, time taken to arrive at a consensus, time taken to apply NOS);

In order to address the above objectives, we will conduct a cross-sectional analytical study on a sample of NRSI publications following this protocol. This protocol will be registered with the Open Science Framework (https://osf.io/). The final study manuscript will be reported according to the STROBE-cross-sectional checklist [15–17]. We plan to report any protocol amendments in the final study manuscript.

### Inter-rater reliability and inter-consensus reliability

Our first objective is to evaluate the inter-rater reliability (IRR) of ROBINS-I at first stage, without customized training and guidance document from the principal investigator, and then at the second stage, with customized training and guidance. At both stages, assessors will have access to the publicly available detailed-guidance developed by the ROBINS-I group. For the second stage, a customized guidance document will be developed using Microsoft word (Word v1.5, Microsoft Corp., Redmond, WA, USA), by a senior member of the team holding Ph.D. degree (MJ). Following review and feedback by another experienced senior member of the team (MA), we will finalize the document. The guidance document will contain simplified decision rules, additional guidance for advanced concepts, as well as clarifications on answering signaling questions that will guide reviewers in making adjudications for each domain in ROBINS-I tool. Once developed, we will send the guidance document to all the reviewers, for help with adjudications in the second stage of the project. Additionally, one training session (via Skype), will be organized by a trainer (MJ) who is a senior member of the team and the developer of the customized guidance document. During the training session, the trainer will review the guidance document with all the reviewers and provide clarifications. We will use the following methods to assess IRR and inter-consensus reliability (ICR).

#### Participating centers

We will involve two pairs of reviewers (LC, NA, RCR, MP, and JX) with varying levels of experience and academic degrees attained, from multiple research teams to assess IRR and ICR. The participating teams are as follows: (coordinating center) The Knowledge Synthesis platform, George and Fay Yee Center for Healthcare Innovation, University of Manitoba (Canada) (MJ, AMAS, LC, NA, RR); Knowledge Synthesis Team, Knowledge Translation Program, Li Ka Shing Knowledge Institute of St. Michael’s Hospital, Unity Health Toronto (Canada) (ACT and RCR); Institute of Health Economics (IHE) (Canada) (MP); and Nottingham Ningbo GRADE Centre (China) (JX).

#### Sample size calculation

We have calculated the sample size (number of NRSI publications) required for IRR assessments (*n* = 44) by taking into account a 5% type I error, 80% statistical power, and an assumed error margin of 30% [18–20]. As suggested by Gwet [18, 19], we assumed the chance-agreement probability (*P*_e_) as 0 (best-case scenario) and estimated the required sample size for IRR using the formulas and calculations available at http://agreestat.com/blog_irr/sample_size_determination.html. We obtained the observed-agreement probability (*P*_a_) between reviewers required for sample size calculation from an initial pilot testing of 10 NRSI publications.

#### Sample selection:

We propose to use a sample of NRSI publications (*n* = 44, based on the sample size calculations) identified through a PubMed (NLM) search of cardiology clinical trials published in English. We will then identify one pre-specified outcome (the primary outcome of each study), for ROB appraisals for each of the included NRSI. With the help of a content expert (SM), we will identify a list of confounders and important co-interventions for the specific association of interest reported in each of the included NRSI publications.

#### Data collection

After the initial pilot testing on 10 studies, we will proceed with ROB assessments for IRR. We will advise the reviewers to review the available general guidelines for ROBINS-I provided by the developers of the ROBINS-I tool available at https://methods.cochrane.org/news/robins-i-tool. We will also advise all reviewers in the participating centers to read the full report of each included NRSI prior to making assessments. Reviewers will have the list of confounders and important co-interventions available during their assessments. At first, two reviewers will independently, and in duplicate, assess the ROB for the included NRSI using the ROBINS-I tool, without using any formal training or customized guidance. For each included NRS, the two reviewers will assess the seven domains of the ROBINS-I tool as *low ROB*, *moderate ROB*, *serious ROB*, *critical ROB*, *or no information* [12] (Table 1). In the end, the two reviewers will resolve conflicts and arrive at a consensus.

**Table 1:** ROBINS-I tool [12]

	Domains	Response options	Support for judgement	Review author’s decision
1.	Bias due to confounding			
1.1	Is there potential for confounding of the effect of intervention in this study?			
	If N/PN to 1.1: the study can be considered to be at low risk of bias due to confounding and no further signaling questions need be considered. If Y/PY to 1.1: determine whether there is a need to assess time-varying confounding:			
1.2	Was the analysis based on splitting participants’ follow-up time according to intervention received?			
	If N/PN, answer questions relating to baseline confounding (1.4 to 1.6) If Y/PY, go to question 1.3			
1.3	Were intervention discontinuations or switches likely to be related to factors that are prognostic for the outcome?			
	If N/PN, answer questions relating to baseline confounding (1.4 to 1.6) If Y/PY, answer questions relating to both baseline and time-varying confounding (1.7 and 1.8)			
1.4	Did the authors use an appropriate analysis method that controlled for all the important confounding domains?			
1.5	If Y/PY to 1.4: Were confounding domains that were controlled for measured validly and reliably by the variables available in this study?			
1.6	Did the authors control for any post-intervention variables that could have been affected by the intervention?			
1.7	Did the authors use an appropriate analysis method that controlled for all the important confounding domains and for time-varying confounding?			
1.8	If Y/PY to 1.7: Were confounding domains that were controlled for measured validly and reliably by the variables available in this study?			
	Risk of bias judgement			
	Optional: What is the predicted direction of bias due to confounding?			
2.	Bias in selection of participants into the study			
2.1	Was selection of participants into the study (or into the analysis) based on participant characteristics observed after the start of intervention			
	If N/PN to 2.1: go to 2.4			
2.2	If Y/PY to 2.1: Were the post-intervention variables that influenced selection likely to be associated with intervention?			
2.3	If Y/PY to 2.2: Were the post-intervention variables that influenced selection likely to be influenced by the outcome or a cause of the outcome?			
2.4	Do start of follow-up and start of intervention coincide for most participants?			
2.5	If Y/PY to 2.2 and 2.3, or N/PN to 2.4: Were adjustment techniques used that are likely to correct for the presence of selection biases?			
	Risk of bias judgement			
	Optional: What is the predicted direction of bias due to selection of participants into the study?			
3.	Bias in classification of interventions			
3.1	Were intervention groups clearly defined?			
3.2	Was the information used to define intervention groups recorded at the start of the intervention?			
3.3	Could classification of intervention status have been affected by knowledge of the outcome or risk of the outcome?			
	Risk of bias judgement			
	Optional: What is the predicted direction of bias due to classification of interventions?			
4.	Bias due to deviations from intended interventions			
	If your aim for this study is to assess the effect of assignment to intervention, answer questions 4.1 and 4.2			
4.1	Were there deviations from the intended intervention beyond what would be expected in usual practice?			
4.2	If Y/PY to 4.1: Were these deviations from intended intervention unbalanced between groups and likely to have affected the outcome?			
	If your aim for this study is to assess the effect of starting and adhering to intervention, answer questions 4.3 to 4.6			
4.3	Were important co-interventions balanced across intervention groups?			
4.4	Was the intervention implemented successfully for most participants?			
4.5	Did study participants adhere to the assigned intervention regimen?			
4.6	If N/PN to 4.3, 4.4 or 4.5: Was an appropriate analysis used to estimate the effect of starting and adhering to the intervention?			
	Risk of bias judgement			
	Optional: What is the predicted direction of bias due to deviations from the intended interventions?			
5.	Bias due to missing data			
5.1	Were outcome data available for all, or nearly all, participants?			
5.2	Were participants excluded due to missing data on intervention status?			
5.3	Were participants excluded due to missing data on other variables needed for the analysis?			
5.4	If PN/N to 5.1, or Y/PY to 5.2 or 5.3: Are the proportion of participants and reasons for missing data similar across interventions?			
5.5	If PN/N to 5.1, or Y/PY to 5.2 or 5.3: Is there evidence that results were robust to the presence of missing data?			
	Risk of bias judgement			
	Optional: What is the predicted direction of bias due to missing data?			
6.	Bias in measurement of outcomes			
6.1	Could the outcome measure have been influenced by knowledge of the intervention received?			
6.2	Were outcome assessors aware of the intervention received by study participants?			
6.3	Were the methods of outcome assessment comparable across intervention groups?			
6.4	Were any systematic errors in measurement of the outcome related to intervention received?			
	Risk of bias judgement			
	Optional: What is the predicted direction of bias due to measurement of outcomes?			
7.	Bias in selection of the reported result			
	Is the reported effect estimate likely to be selected, on the basis of the results, from…			
7.1	…multiple outcome measurements within the outcome domain?			
7.2	…multiple analyses of the intervention-outcome relationship?			
7.3	…different subgroups?			
	Risk of bias judgement			
	Optional: What is the predicted direction of bias due to selection of the reported result?			
	Overall risk of bias			
	Risk of bias judgement			
	Optional: What is the overall predicted direction of bias for this outcome?			

As a next step, each pair of reviewers will re-assess the same set of NRSI following formal training and using a customized guidance sheet following the initial “without guidance” ROB assessments. At the end of the assessments, again the reviewers will meet to resolve conflicts and arrive at a consensus. All studies are assessed first without guidance, before any with-guidance assessments, to prevent the possibility of with-guidance assessment influencing without-guidance assessment. The principal investigator (MJ) at the coordinating center will coordinate this process among reviewers in the different participating centers.

Upon completion, the collaborating center will collect, organize, and transfer the ROB assessment data from various reviewers to an Excel workbook, prior to proceeding with the data analysis. We will then assess and report the IRR and ICR for ROB assessments “without guidance,” and “with guidance,” separately.

#### Data analysis

An experienced biostatistician (RR) from the collaborating center will conduct all the analyses in collaboration with the other members of the research team. We will transfer all collected data from the Microsoft Excel workbook (Excel v14, Microsoft Corp., Redmond, WA, USA) to SAS (9.4), (SAS Institute Inc., Cary, NC, USA) for analysis. The kappa (κ) statistic is typically used to assess IRR as it corrects for the “chance” agreement between the two reviewers and allows for different types of disagreements to have differing weights [21]. The chance-agreement probability evaluated by the κ statistic assumes that all observed ratings may yield agreements by chance, thus leading to unpredictable results in the presence of high agreement between reviewers [22]. The AC_1_ statistic developed by Gwet [22] calculates the true overall chance agreement in the presence of high agreement reviewers, thus yielding values closer to “true” IRR [23]. We will also analyze inter-consensus reliability (ICR) using Gwet’s AC_1_ statistic [22].

The agreements among reviewers (IRR and ICR) will be categorized as follows [24]: poor (0), slight (0.1–0.2), fair (0.21–0.4), moderate (0.41–0.6), substantial (0.61–0.8) or near-perfect (0.81–0.99). We will tabulate the AC_1_ values and the 95% confidence intervals (CIs) separately (without or with guidance), as shown in Table 2. Additionally, we will assess the correlations between adjudications made during both the stages (“with guidance” and “without guidance”) for each of the reviewers to ensure that the effect of training and guidance is not biased.

**Table 2:** Reporting of IRR & ICR for ROBINS-I (with or without guidance)

						IRR		ICR	
						Without customized guidance	With customized guidance	Without customized guidance	With customized guidance
Bias domains	ROB assessments					AC_1_ (95% CI)	AC_1_ (95% CI)	AC_1_ (95% CI)	AC_1_ (95% CI)
	L	M	S	C	NI				
Confounding									
Selection of participants									
Classification of interventions									
Departures from intended interventions									
Missing data									
Measurement of outcomes									
Selection of reported results									
Overall									

*L* low, *M* moderate, *S* serious, *C* critical, *NI* no information

### Concurrent validity

The second objective of this study is to evaluate the concurrent validity of the ROBINS-I compared to NOS. Concurrent validity refers to how well a newly developed tool is correlated to similar domains of a widely used tool at the same point in time [25]. In other words, concurrent validity evaluates the extent to which there is concordance in judgment for similar domains in both the tools that are being compared [25]. Currently, there is no “gold standard” tool to asses ROB in NRSI. Hence, to assess the concurrent validity of ROBINS-I, we propose to use NOS, as it the most commonly used quality assessment tool for NRSI that had been previously recommended by Cochrane [26].

In this cross-sectional study, we will explore the concordance between assessments made on similar domains in ROBINS-I and NOS, and the overall assessments for each included NRS.

#### Data collection

As mentioned previously, we will use a sample of NRS (*n* = 44) for assessments of concurrent validity. We have compared and matched both NOS and ROBINS-I (as shown in Tables 3 and 4) to identify the items that *completely overlap*, *partially overlap*, or *unique* to each tool. Since the theoretical construct differs between NOS (methodological quality) and ROBINS-I (ROB), we did not expect a complete match between all domains.

**Table 3:** Comparison of domains between NOS^7^ and ROBINS-I [12]

NOS		ROBINS-I		Degree of overlap
Comparability	*C: Comparability of cohorts on the basis of the design or analysis*	Bias due to confounding	1.1: Is there potential for confounding of the effect of intervention in this study?	Unique
	*1a: Study controls for the most important factor*		1.2: Was the analysis based on splitting participants’ follow-up time according to intervention received?	Unique
	*1b: Study controls for additional factor*		1.3. Were intervention discontinuations or switches likely to be related to factors that are prognostic for the outcome?	Unique
		*Baseline confounding only*	*1.4. Did the authors use an appropriate analysis method that controlled for all the important confounding domains?*	Complete overlap
			1.5: Were confounding domains that were controlled for measured validly and reliably by the variables available in this study?	Unique
			1.6. Did the authors control for any post-intervention variables that could have been affected by the intervention?	Unique
		*Time-varying confounding only*	1.7: Did the authors use an appropriate analysis method that controlled for all the important confounding domains and for time-varying confounding?	Unique
			1.8: Were confounding domains that were controlled for measured validly and reliably by the variables available in this study?	Unique
Selection	S1: Representativeness of exposed cohort	Bias in selection of participants into the study	2.1. Was selection of participants into the study (or into the analysis) based on participant characteristics observed after the start of intervention?	Unique
	*1a: Truly representative*		2.2: Were the post-intervention variables that influenced selection likely to be associated with intervention?	Unique
	*1b: Somewhat representative*		2.3: Were the post-intervention variables that influenced selection likely to be influenced by the outcome or a cause of the outcome?	Unique
	*1c: Selected group of users*		2.4. Do start of follow-up and start of intervention coincide for most participants?	Unique
	*1d: No description of the derivation of the cohort*		2.5: Were adjustment techniques used that are likely to correct for the presence of selection biases?	Unique
	S2: Selection of non-exposed cohort			
	*2a: Drawn from the same community as the exposed cohort*			
	*2b: Drawn from a different source*			
	*2c: No description of the derivation of the non-exposed cohort*			
Selection	S3: Ascertainment of exposure	Bias in classification of interventions	3.1 Were intervention groups clearly defined?	Unique
	*3a: Secure record*		3.2 Was the information used to define intervention groups recorded at the start of the intervention?	Unique
	*3b: Structured interview*		*3.3 Could classification of intervention status have been affected by knowledge of the outcome or risk of the outcome?*	Partial overlap
	*3c: Written self-report*			
	*3d: No description*			
	*S4: Demonstration of outcome of interest was not present at start of the study*			
	*4a: Yes*			
	*4b: No*			
		Bias due to deviations from intended interventions	4.1. Were there deviations from the intended intervention beyond what would be expected in usual practice?	Unique
			4.2: Were these deviations from intended intervention unbalanced between groups and likely to have affected the outcome?	Unique
			4.3: Were important co-interventions balanced across intervention groups	Unique
			4.4: Was the intervention implemented successfully for most participants?	Unique
			4.5: Did study participants adhere to the assigned intervention regimen?	Unique
			4.6: Was an appropriate analysis used to estimate the effect of starting and adhering to the intervention?	Unique
Outcomes	O1: Was follow-up long enough for outcomes to occur (Yes/No)	Bias due to missing data	*5.1: Were outcome data available for all, or nearly all, participants?*	Partial overlap
	*O3: Adequacy of follow-up of cohorts*		5.2: Were participants excluded due to missing data on intervention status?	Unique
	*3a: Complete follow-up -all subjects accounted for*		5.3: Were participants excluded due to missing data on other variables needed for the analysis?	Unique
	*3b: Subjects lost to follow-up unlikely to introduce bias -small number lost*		*5.4: Are the proportion of participants and reasons for missing data similar across interventions?*	Partial overlap
	*3c: Follow-up rate large (%) and no description of those lost*		5.5: Is there evidence that results were robust to the presence of missing data?	Unique
	*3d: No statement*			
Outcomes	*O2: Assessment of outcome*	Bias in measurement of outcomes	*6.1 Could the outcome measure have been influenced by knowledge of the intervention received?*	Partial overlap
	*2a: Independent blind assessment*		*6.2 Were outcome assessors aware of the intervention received by study participants?*	Partial overlap
	*2b: Record linkage*		*6.3 Were the methods of outcome assessment comparable across intervention groups?*	Partial overlap
	*2c: Self report*		6.4 Were any systematic errors in measurement of the outcome related to intervention received?	Unique
	*2d: No description*			
		Bias in selection of the reported result	7.1: Is the reported effect estimate likely to be selected, on the basis of the results, from multiple outcome measurements within the outcome domain?	Unique
			7.2: Is the reported effect estimate likely to be selected, on the basis of the results, from multiple analyses of the intervention-outcome relationship?	Unique
			7.3: Is the reported effect estimate likely to be selected, on the basis of the results, from different subgroups?	Unique

The entries in italics are items in both tools that either completely or partially overlap

The entries that are upright are items that are unique to each tool

**Table 4:** Similar items between NOS [7] and ROBINS-I [12] for various domains

	Similar domains	ROBINS-I (signaling questions)	NOS (domain items)	Degree of overlap
1.	ROBINS-1: bias due to confounding NOS: comparability	1.4	C1a, C1b	Complete overlap
2.	ROBINS-I: bias in selection of participants NOS: selection	–	–	Unique
3.	ROBINS-I: bias in classification of interventions NOS: demonstration of outcome of interest was not present at start of the study	3.3	S4a, S4b	Partial overlap
4.	ROBINS-I: bias due to deviations from intended interventions NOS: –	–	–	Unique
5.	ROBINS-I: bias due to missing data NOS: adequacy of follow-up of cohorts	5.1, 5.4	O3a, O3b, O3c, O3d	Partial overlap
6.	ROBINS-I: bias in measurement of outcomes NOS: assessment of outcome	6.1, 6.2, 6.3	O2a, O2b, O2c, O2d	Partial overlap
7.	ROBINS-I: bias in selection of the reported result NOS: –	–	–	Unique

For the assessment of concurrent validity, one reviewer (MJ) with expertise in systematic reviews will assess NOS on a sample of NRSI (*n* = 44). We will then compare these NOS adjudications with the after-consensus ROBINS-I adjudications (done after customized training and guidance by two pairs of reviewers) for the same set of studies that were used for the ICR assessments.

We will calculate the correlation between the two tools for each of the domains and for the overall assessments. For comparison of overall assessments between the two tools, we will use the following algorithm: 0–2 stars in NOS will be considered similar to “critical ROB” in ROBINS-I, 3–5 stars in NOS will be considered as similar to “serious ROB” in ROBINS-I, 6–8 stars in NOS will be considered as similar to “moderate ROB” in ROBINS-I, and 9 stars in NOS will be considered as similar to “low ROB” in ROBINS-I. In addition, for any discordance observed between domains or overall assessment, we will explore the possible reasons and attempt to provide explanations.

#### Data analysis

An experienced biostatistician (RR) from the collaborating center will conduct all the analyses in collaboration with the other members of the research team. We will transfer all collected data from the Excel workbook to SAS (9.4), (SAS Institute Inc., Cary, NC, USA) for analysis.

We will use the following algorithm for comparison between similar items (partially or completely overlapping) in the two tools (NOS and ROBINS-I):

1. *For the “selection” domain in NOS*: assessments with four stars will be considered equivalent to “low ROB” adjudication in ROBINS-I. Assessments with three stars will be considered equivalent to “moderate ROB” adjudication in ROBINS-I. Assessments with two stars will be considered equivalent to “serious ROB” adjudication in ROBINS-I, and assessments with zero or one star will be considered equivalent to “critical ROB” adjudication in ROBINS-I.
2. *For the “comparability” domain in NOS*: assessments with two stars will be considered equivalent to “low ROB” adjudication in ROBINS-I. Assessments with one star will be considered equivalent to “moderate ROB” adjudication in ROBINS-I. Assessments with zero star will be considered equivalent to “serious or critical ROB” adjudication in ROBINS-I.
3. *For the “outcome assessment” domain in NOS*: assessments with three stars will be considered equivalent to “low ROB” adjudication in ROBINS-I. Assessments with two stars will be considered equivalent to “moderate ROB” adjudication in ROBINS-I. Assessments with one star will be considered equivalent to “serious ROB” adjudication in ROBINS-I, and assessments with zero star will be considered equivalent to “critical ROB” adjudication in ROBINS-I.
4. The NOS domains with “no description/no statement” assessments will be considered equivalent to the “no information” adjudication in ROBINS-I.

For measuring concordance or discordance between various domains of NOS and ROBINS-I (i.e., to assess the concurrent validity of ROBINS-I), we will use “Kendall’s tau” a rank correlation coefficient statistic [27], and its 95% confidence intervals (for ordinal variables) for each domain and for the overall assessments.

### Evaluator burden

The time taken to apply any newly developed instrument is an important factor to consider, as it may contribute to a significant burden on the evaluator/reviewer. It is also important to assess factors that could reduce the application time. In this study, we will compare the time taken to apply ROBINS-I (without and with guidance), time taken by the reviewer pairs to arrive at a consensus (without and with guidance), and the time taken to apply NOS for comparison with ROBINS-I.

#### Data collection process

Reviewers will record (using a digital clock) the time taken (in minutes) while applying (time to read article plus time to adjudicate) ROBINS-I tool (without and with guidance), time taken for consensus, and the time taken to apply the NOS tool (time to read article plus time to adjudicate) for each included NRS. The reviewers will use the Excel workbook created by the principal investigator to record the start time, end time, and total time to apply ROBINS-I at the completion of the assessment for each NRS and after the consensus process with the second reviewer. The reviewers will split the time to apply ROBINS-I into the time taken to read the full-text of the NRS and the time taken for adjudications. The time to apply ROBINS-I will begin when the reviewer begins reading the full-texts of the NRS and will end when decisions for all domains are completed and an overall ROB assessment for the study is established. The average overall time to apply ROBINS-I for the same set of articles assessed by each reviewer will be calculated. In addition, we will also calculate the time taken to resolve conflicts and arrive at a consensus, and the overall time (time to apply plus time taken to arrive at a consensus) for each pair of reviewers. The time to arrive at a consensus will start when the two reviewers convene to resolve conflicts and will end when they arrive at a consensus.

#### Data analysis

An experienced biostatistician (RR) from the coordinating center will conduct all the analyses in collaboration with the other members of the research team. We will transfer all collected data from the Excel workbook to SAS (9.4), (SAS Institute Inc., Cary, NC, USA) for analysis.

1. We will first summarize the average time (mean and SD) taken by the reviewers to assess ROBINS-I without guidance and with guidance separately.
2. To analyze the impact of customized training and guidance on changes in evaluator burden (ROBINS-I assessment time as well as the time taken by the reviewer pairs to arrive at consensus), we will compare two centers separately (*n* = 44 respectively). We will use generalized linear models to evaluate changes in the time taken to assess ROBINS-I after customized guidance (compared to without guidance). We will control for the correlation between reviewers using random effects. The distribution of the outcome will be adjusted by using a link function.
3. To analyze the time taken to apply ROBINS-I compared to NOS we will use a fixed effect, generalized linear model. The model distribution will be chosen by link function.

## Discussion

Systematic reviews that include NRS can provide valuable evidence on rare outcomes, adverse events, long-term outcomes, real-world practice, and in situations where RCTs are unavailable [12, 28]. It is very important to appraise the risk of bias in the included NRS to have a complete understanding of the strengths and weaknesses of the overall evidence, as methodological flaws in the design or conduct of the NRS could lead to biased effect estimates [12]. The newly developed ROBINS-I could be a very useful tool for researchers in assessing the risk of bias in NRS when undertaking systematic reviews of NRS. As such, it is important to evaluate the usability, reliability, and concurrent validity of this tool to help identify potential barriers and facilitators in applying this tool in a real-world setting.

In this cross-sectional study protocol, we describe the methods we will use to assess the inter-rater reliability, inter-consensus reliability, and the concurrent validity of ROBINS-I. Our proposed study, upon completion, will provide empirical evidence on the IRR, concurrent validity, and the evaluator burden of ROBINS-I.

### Strengths and challenges

Across the world, researchers, with a range of expertise, conduct systematic reviews that include NRSI. ROBINS-I tool was designed to be used by all systematic reviewers with varied academic backgrounds and experience. A major strength of our study is that we will involve reviewers from multiple research teams with a range of expertise and academic backgrounds (highest degree attained) to apply and test ROBINS-I, in order to simulate the real-world settings. We will also use a sample of NRS that were not evaluated previously by the reviewers, in order to mimic what is typically encountered in a real-world setting. As with any elaborate tool, it is important to evaluate concerns regarding the practical use of ROBINS-I. To the best of our knowledge, there are two studies [29, 30] that have assessed the IRR of ROBINS-I. In Losilla et al. [29], using a sample of studies on health psychology, the IRR for ROBINS-I was reported to range from slight to an almost perfect agreement for various items and domains using the kappa statistic. Whereas in Minozzi et al. [30], using Fleiss’ Kappa statistic, the IRR for ROBINS-I for all domains were reported to be only of slight agreement. To the best of our knowledge, there are no studies that have assessed the impact of additional training/guidance on IRR, the impact of additional training/guidance on evaluator burden, the ICR, the impact of additional training/guidance on the ICR, and also the construct validity of ROBINS-I (comparison of ROBINS-I with NOS).

The findings of our cross-sectional study have a potential to elucidate the impact of training and development of customized guidance with decision rules on the IRR, ICR, and the evaluator burden of ROBINS-I. Also, for data analysis, we will use the AC_1_ statistic developed by Gwet [22] to calculate true chance agreement in the presence of high agreement between reviewers, thus yielding values closer to “true” IRR for ROBINS-I.

For feasibility, the reviewers will only appraise ROB for a single outcome for each NRSI. This may be a limitation as reviewers in real-world settings may need to appraise multiple outcomes for each of the included NRSI and the evaluator burden might differ slightly from the findings of this study. In addition, we anticipate that the time taken to assess ROB might be longer for NRSI appraised at the beginning compared to those appraised later, due to increasing familiarity and a learning curve. In a real-world setting, the training and customized guidance decision rules developed by the researchers for their own systematic reviews may differ from the one developed by the principal investigator of this study, and this may pose a challenge in the generalization of the findings of this study. For feasibility, we have proposed to use the same reviewers for both stages (without and with guidance), and we anticipate that this may bias the effect of training and guidance. However, we will address this limitation by assessing the correlations between adjudications made during the two stages, for each of the reviewers. A poor correlation between adjudications made during the two stages, for a reviewer would indicate that the training and guidance have been useful.

As with any new tool, it is critical to assess the IRR, ICR, concurrent validity, and evaluator burden of ROBINS-I, in order to improve the consistency of its application and its interpretation across various systematic reviews that include NRS. We hope that the findings of this study will contribute to an improved understanding and better application of the ROBINS-I tool.

### Knowledge dissemination strategy

Systematic reviews serve as a source of knowledge and evidence to aid in the decision-making process. Our cross-sectional study addresses issues that may contribute to the quality of the evidence synthesized by the systematic review and thus will be of great interest to all stakeholders such as clinicians, decision-makers, patients, and the general public. It will also be of great interest to researchers to improve their understanding regarding the practical use of the ROBINS-I tool. We plan to disseminate the results of our cross-sectional study by presenting the study results at various conferences, by publishing study results in academic journals and by spreading the message through social media.

## Data Availability

Not applicable.

## Abbreviations

ACROBAT-NRSI: A Cochrane Risk of Bias Assessment Tool for Non-Randomized Studies of Interventions
ICR: Inter-consensus reliability
IRR: Inter-rater reliability
NLM: National Library of Medicine
NOS: Newcastle-Ottawa scale
NRS: Non-randomized studies
NRSI: Non-randomized studies of interventions
RCT: Randomized controlled trials
ROB: Risk of bias
ROBINS-I: Risk of Bias in Non-randomized Studies of Interventions
SD: Standard deviation
κ: Kappa statistic
